# Investigating the suitability of poly tetraarylphosphonium based anion exchange membranes for electrochemical applications

**DOI:** 10.1038/s41598-021-93273-x

**Published:** 2021-07-05

**Authors:** Muthumeenal Arunachalam, Alessandro Sinopoli, Farida Aidoudi, Stephen E. Creager, Rhett Smith, Belabbes Merzougui, Brahim Aïssa

**Affiliations:** 1 Qatar Environment and Energy Research Institute, P.O. Box: 34110, Doha, Qatar; 2 Department of Chemistry and Center for Optical Materials Science and Engineering Technology, Clemson University, Clemson, SC USA

**Keywords:** Chemistry, Materials science

## Abstract

Anion exchange membranes (AEMs) are becoming increasingly common in electrochemical energy conversion and storage systems around the world (EES). Proton-/cation-exchange membranes (which conduct positive charged ions such as H^+^ or Na^+^) have historically been used in many devices such as fuel cells, electrolysers, and redox flow batteries. High capital costs and the use of noble metal catalysts are two of the current major disadvantages of polymer electrolyte membrane (PEM)-based systems. AEMs may be able to overcome the limitations of conventional PEMs. As a result, polymers with anion exchange properties have recently attracted a lot of attention due to their significant benefits in terms of transitioning from a highly acidic to an alkaline environment, high kinetics for oxygen reduction and fuel oxidation in an alkaline environment, and lower cost due to the use of non-precious metals. The aim of this research was to learn more about the development of a new AEM based on poly tetraarylphosphonium ionomers (pTAP), which has high ionic conductivity, alkaline stability, thermal stability, and good mechanical properties, making it a more cost-effective and stable alternative to conventional and commercial AEMs. A simple solution casting method was used to build novel anion exchange composite membranes with controlled thicknesses using the synthesized pTAP with polysulfone (PS). To ensure their suitability for use as an electrolyte in alkaline electrochemical systems, the composite membranes were characterized using FTIR, XRD, water uptake, ionic conductivity, and alkaline stability. At 40 °C, the PS/pTAP 40/60 percent membrane had a maximum ionic conductivity of 4.2 mS/cm. The thermal and mechanical stability of the composite membranes were also examined, with no substantial weight loss observed up to 150 °C. These findings pave the way for these membranes to be used in a wide variety of electrochemical applications.

## Introduction

The increased awareness of environmental issues and growing demand for sustainable energy sources have led to accelerated research efforts in the field of energy conversion and storage systems^1^. In this regard, Redox Flow Batteries (RFBs) are nowadays gaining momentum due to their suitability for large storage applications^2^. Most electrochemical conversion and storage systems are dependent on ion-exchange membranes. Cation exchange membranes (CEMs) have attracted tremendous attention owing to their high ion conductivity and chemical stability. However, applications of CEMs suffer from several limitations, such as the requirement of noble metal-based catalyst (e.g. Pt) and high crossover of fuels or positively charged redox species. Despite great success, there are still many challenges to be addressed. These challenges can be mitigated with the use of suitable anion exchange membranes (AEMs).

Polymers with anion exchange properties are currently gaining a lot of popularity. In reality, when AEMs are used, precious metal catalysts are no longer needed, and transition metal catalysts can be used instead. This will promote in parallel: (i) the facile oxidation of hydrogen or alcohol at the anode under alkaline conditions^3,4^, (ii) in an alkaline environment, high kinetics for oxygen reduction and fuel oxidation, and (iii) the restriction of positive species crossover from the anode, which is usually very quick in CEMs due to the opposite migration of hydroxide ions from the cathode to the anode^5,6^.

Many research efforts have been centered on the production of AEMs in recent years, which have primarily been based on polystyrene cross-linked with divinylbenzene with the quaternary ammonium group linked to a benzylic methylene group. Because of their low cost and ease of synthesis, these polymers were used in early studies^7^. They do, however, have a range of disadvantages, including low chemical and thermal stability, as well as reduced processability. Many other polymers such as polyarylene sulfone^8^, poly-phenylene oxide (PPO), polyetherimide, polyether ether ketone (PEEK)^9^, polybenzimidazole^10^, copolymers from vinyl monomers, and grafted fluoropolymers, have been developed as promising alternatives to address these issues, and have been successfully employed as PEM in energy conversion and storage systems, under strongly alkaline and oxidative conditions and at high temperatures^10^. Under such extreme conditions, however, none of the commercial AEMs displayed sufficient chemical stability.

Our objective was then to develop alkali stable anion exchange separator, resisting to some forms of decomposition under very strong alkaline conditions. Because organo-phosphonium cations are promising yet much less-studied candidate for alkaline ionomers compared to other quaternary nitrogen or sulphur based organocations^11–15^, we focused hence on developing a new generation of AEMs based on tetraaryl-phosphonium ionomers (pTAP). In fact, upon exposure to concentrated alkali, most simple phosphonium salts decompose in a similar manner to ammonium salts by a combination of nucleophilic attack, proton abstraction or direct attack of hydroxide on phosphorus^11,16–18^. However, tetra aromatic groups attached to phosphorus are much less susceptible to base or nucleophilic attack due to the lack of protons on aliphatic carbons, and they can provide electronic and steric stabilization of the phosphonium center. Based on these considerations, we shed light on the synthesis and formulating methods for developing AEMs based on phosphonium class of polymers. We report on their ionic conductivity along with high alkaline stability, thermal stability, and good mechanical properties, which provide a pathway for the significant advancement over the state of the art AEMs based on nitrogen functional groups.

## Results

**Figure 1:**
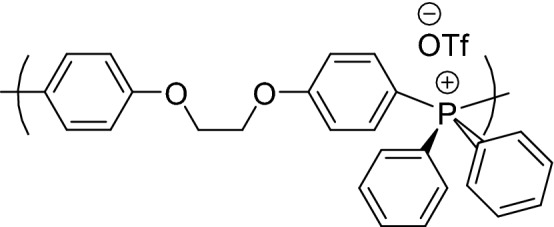
The structure of tetraarylposphonium polymer^19^.

Wan et al*.* have explored various methods to synthesize pTAP polymers^19^. The structure of the synthesized pTAP polymer is shown in Fig. 1, the detailed synthesis is reported in the ESI. The chemical structure of the polymer was confirmed by ^1^H, ^19^F and ^31^P NMR analysis (see ESI). The proton NMR confirmed the presence of aromatic protons in the region between 7.2 and 8.1 ppm, whereas the alkyl protons lie at 4.4 ppm. In the ^31^P NMR spectrum, the main phosphonium peak was observed at 23.9 ppm, minor peaks attributed to the phosphine oxide end groups were observed between 25.3 and 25.7 ppm. ^19^F spectrum shows one dominant peak at -78.82 ppm assigned to the triflate groups of the polymer backbone.

The triflate anion (CF_3_SO_3_^-^), abbreviated as OTf, can be replaced with any desirable anion including OH^-^. Initially, 20 wt. % of polysulfone polymer pellets were dissolved in *N*-Methyl-2-pyrrolidone solvent with continuous stirring at 40 °C until a homogenous solution was obtained. The casting dope was prepared by adding various appropriate weight ratios (40–70 wt.%) of pTAP ionomer polymer to the already dissolved PS solution and the total polymer solution is 20 wt. %. The resulting solution is stirred continuously for 24 h until the blend turned into a homogenous solution. The required weight percent of pTAP is restricted to 70 wt.% due to the aggregation of particles in the polysulfone matrix at higher weight percentages.

### Fourier transform infra-red spectroscopy

**Figure 2:**
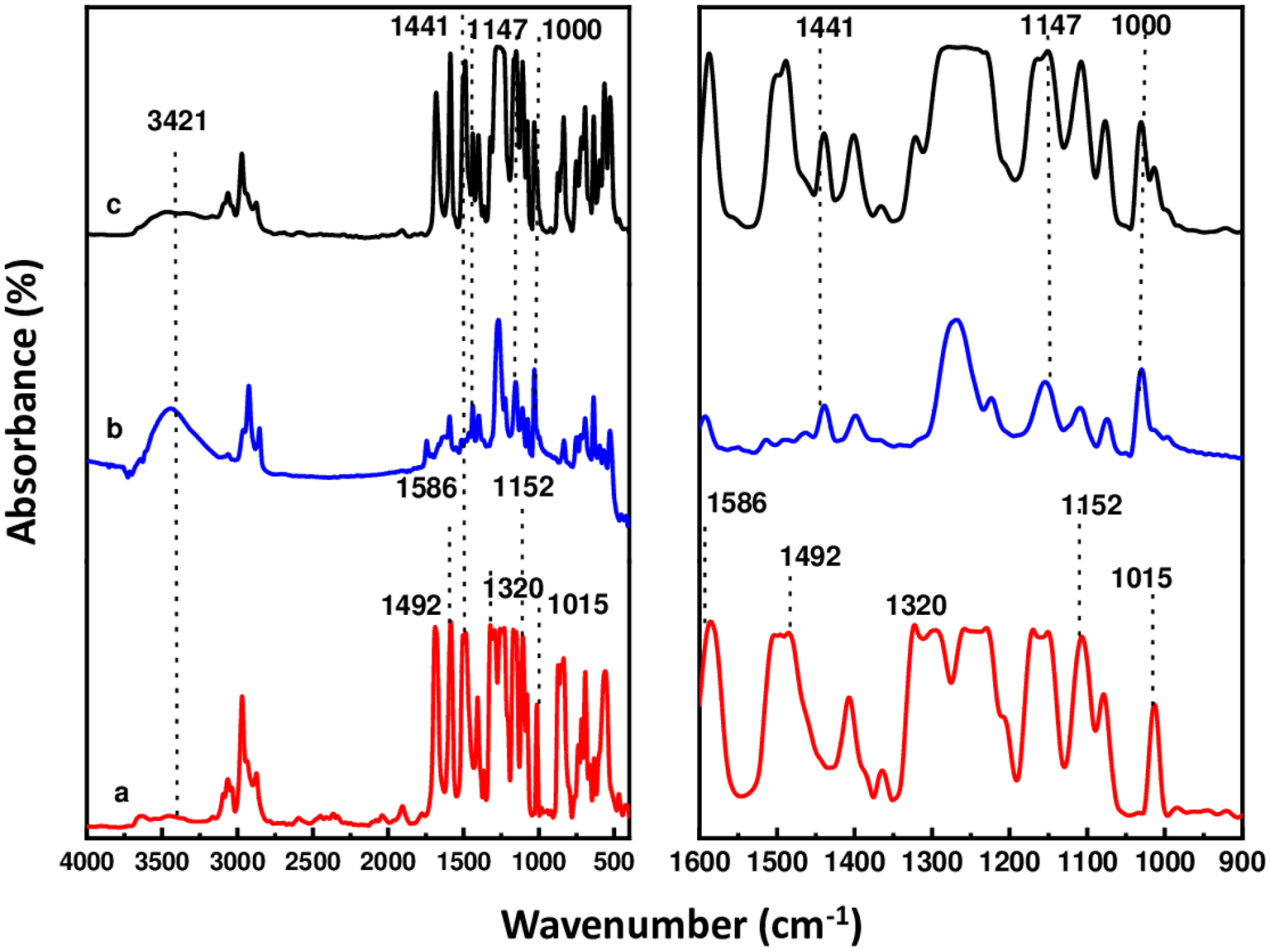
FTIR spectra of membranes **(a)** polysulfone, **(b)** tetraarylphosphonium polymer, **(c)** PS/pTAP (40/60%) composite membranes.

The FTIR spectra of pure pTAP polymer powder, polysulfone and the fabricated composite blend of PS/pTAP are shown in Fig. 2 along with the expanded spectra. Peaks for polysulfone have been assigned by comparison with previously reported analysis^20^. The main absorption bands in Fig. 2a are: 1492 and 1586 cm^−1^ (C–C in-ring vibration); 1320 and 1297 cm^−1^ (O=S=O asymmetric stretching vibration); 1233 cm^−1^ (C–O stretching vibration); 1163 and 1152 cm^−1^ (O=S=O symmetric vibration) and 1015 cm^−1^ (aryl groups). In Fig. 2c, the spectra contain all the group frequencies corresponding to the phenyl group even though it is attached to the phosphorous compound. In addition, there are strong absorption bands near 1000 cm^−1^, and 1147 cm^−1^, 1441 which are due to the phenyl group attached directly to phosphorus. The broad absorption band at 3421 cm^−1^ in Fig. 2c is due to the free hydroxyl group present in the compound and in Fig. 2b it might due to the moisture content in the polymer. Hence it is confirmed that the conversion of triflate ions into –OH has been taken place after immersion in KOH solution. The characteristic peaks of the pTAP polymer and polysulfone are present in the blended membranes.

### Ion exchange capacity

It has been widely reported in the literature that the hydroxyl ion conductivity of AEMs is associated with the IEC of the membranes^21^. As a result, increasing the IEC will increase ionic conductivity. Increases in the IEC alone, on the other hand, can result in an excess of water absorption and a large swelling ratio, resulting in dimensional instability. The IEC of the composite blends increased as the weight fraction of pTAP polymer in the composite blend increased in our samples. The IEC values of the fabricated AEMs are in reasonable agreement with literature and they range from 0.95 meq/g for PS/pTAP (60%/40%), to 1.11 meq/g PS/pTAP (50%/50%), up to 1.2 meq/g for PS/pTAP (40%/60%). In order to achieve high IEC, we tried to increase the weight percentage of quaternary phosphonium ion exchange groups beyond 70 wt. % in polysulfone matrix, phase segregation occurred resulting in in-homogeneous membrane. During the membrane forming process with the high amount of pTAP polymer in the polysulfone matrix, we assisted to phase separation and individual precipitation.

### Hydroxyl ion conductivity of the composite blend

**Figure 3:**
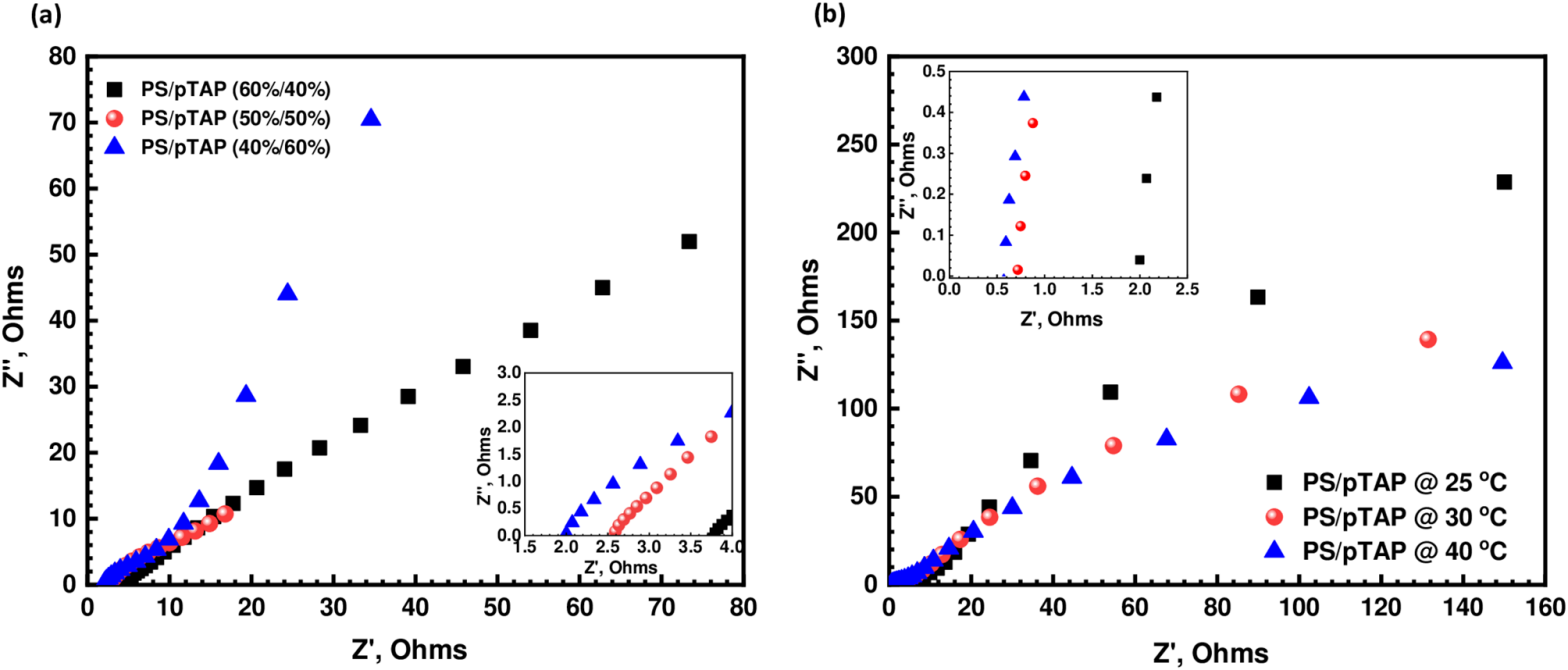
**(a)** Nyquist plots of PS/pTAP composite membranes with various weight loadings. **(b)** Nyquist plot for PS/pTAP (40%/60%) composite membrane with respect to temperature.

The ionic conductivity of the composite membranes was analyzed by running electrochemical impedance spectroscopy (EIS) method using potentiostat Biologic SP300. Generally, hydroxyl ion conductivity of AEM is comparatively lower than that of the proton conductivity of PEM due to its difference in size and mobility. In this study, all the composite membranes exhibited favorable conductivities within a range of 0.8 to 4.2 mS/cm, which could meet the requirement for usage. The Nyquist plots of the composite membrane with different weight loadings and with respect to temperature were shown in Fig. 3a,b. The information of bulk resistance can be obtained from the typical AC impedance spectra of composite membranes and it showed the dependence of hydroxyl ion conductivity on ionomer content of the fabricated composite blend membranes. Hence we increased the pTAp ionomer in order to enhance the ionic conductivity from 40 to 60% in the polysulfone matrix. This can be attributed to the increasing number of ion exchange groups and also the water content, which facilitate the ionic transport^22,23^. The hydroxyl ion conductivity increases from 0.8 mS/cm to 1.8 mS/cm with the increasing content of phosphonium ionomer counterpart in the composite blend membrane. The phase segregation morphological structure of Nafion (e.g., proton form) is commonly credited with its outstanding ionic conductivity. The existence of both a strongly hydrophobic fluorocarbon polymer backbone and flexible side chains (that contain ionic groups, sulfonic group) results in the creation of a wider ionic channels between hydrophilic/hydrophobic phase, in which ion-containing hydrophilic domains overlap and form interconnected ionic channels^24,25^. Even though the OH^-^ conduction follows a similar mechanism to H^+^ conduction^26^, the AEMs have lesser ionic conductivity. This might be due to the development of narrow ion-conducting channels between the less hydrophobic polysulfone and also the less hydrophilic pTAP polymer. It is possible to improve considerably the ionic conductivity through the adequate hydrophilicity of polymer electrolytes which in truns leads to higher ion transport properties in the membranes.

**Figure 4:**
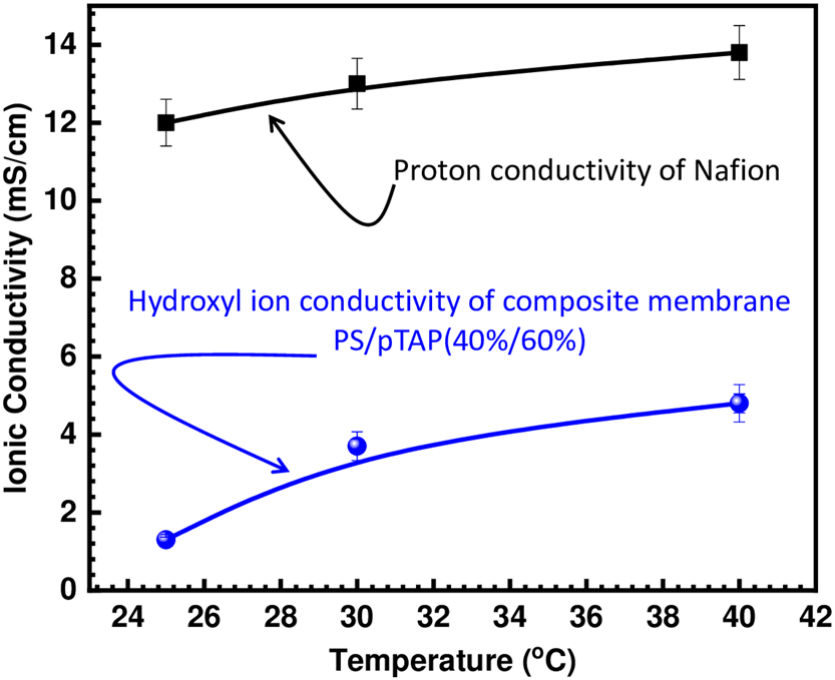
Comparison of ionic conductivity of PS/pTAP (40%/60%) composite membrane with Nafion 212.

Figure 4 depicts the composite membranes' hydroxyl ion conductivity as a function of temperature. The membranes' hydroxide ion conductivity increases as the temperature rises. The maximum hydroxide ion conductivity of the PS/pTAP (40%/60%) membrane at 40 °C was 4.2 mS/cm. This is due to the fact that ions have a higher diffusivity and migrate faster at higher temperatures. Furthermore, at high temperatures, the polymer matrix became more flexible and more water was adsorbed to the membrane, resulting in higher ionic conductivity^27^. The proton conductivity of Nafion is three times higher than that of the hydroxyl ion, as shown in Fig. 4. The anion conductivity of AEMs may be limited by the poor basicity of the quaternary phosphonium groups combined with the lower mobility of the OH^-^ compared to H^+^ (ion mobility in dilute solution of H^+^  = 4.76 and of OH^-^ = 2.69 relative to K^+^^28^). In aqueous solutions, both H^+^ and OH^-^ transport exhibited Grotthuss activity^29^. Hydroxyl anions, on the other hand, have stable solvation shells that reorganize the solvent molecules and disrupt the hydrogen bond network, while hydronium ions are normally incorporated into the hydrogen bond network of water. The ratio between diffusion coefficients of protons (D_H_^+^  = 9.3 × 10^−9^m^2^ s^−1^) and hydroxyl ions (D_OH_^−^  = 5.3 × 10^−9^ m^2^ s^−1^), measured in liquid water at 25 °C^30^, is about 1.8, the ratio between the conductivity of Nafion and AEM based on PS/pTAP composites (Fig. 4) is almost double. Eventhough our fabricated membranes have shown a lower ionic conductivity compared to that of the commercial AEMs, including Fumasep-FAA3 (40 mS/cm), and AEMION-AF1-HNN5-50-X (15–25 mS/cm))^31^, however, our reported values are closely comparable to those reported in the relevant literature. As a matter of fact, the hydroxyl ion conductivities of quaternized/cross-linked PVA-chitosan^32^, PVA-poly (acrylonitrile-co-2-dimethy laminoethyl methacrylate)^33^ and trimethylammonium poly (ether imide)^34^ have been reported in the range of 3–7 mS/cm, 3.45 mS/cm, and 2.28–3.51 mS/cm, respectively.

### Water uptake

The presence of water in the hydrophilic domains promotes the transport of OH^-^ ions, water uptake is an important parameter in studying AEMs. Higher ionic conductivity is caused by a greater amount of adsorbed water molecules solvating the polymer moieties to a greater degree. A high water uptake, on the other hand, can result in a loss of mechanical stability.

Since the presence of water is needed for the conduction of ions such as OH^-^, the structure of hydrophilic domains in a polymer electrolyte is the most important factor in ion conduction. Table 1 shows the water absorption values of composite blend membranes at ambient and 60 °C. Water uptake values increased as the weight fraction of pTAP ionomer in the composite blend membrane increased, as predicted. The increase in incorporated hydrophilic pTAP ionomer materials into the composite blend membranes may be responsible for the increased water absorption ability. The water absorption of the fabricated membranes, on the other hand, increased with temperature, which is due to the increased mobility of polymer chain segments and the increased free volume of the polymer at high temperatures.

**Table 1:** Water uptake for the fabricated PS/pTAP composite membranes.

Composite blend/PS/pTAP wt.%	Water uptake at RT/ %	Water uptake at 60 °C / %
60/40	5.4	7.8
50/50	5.6	9.4
40/60	8.6	10.3

### Contact angle measurements

**Figure 5:**
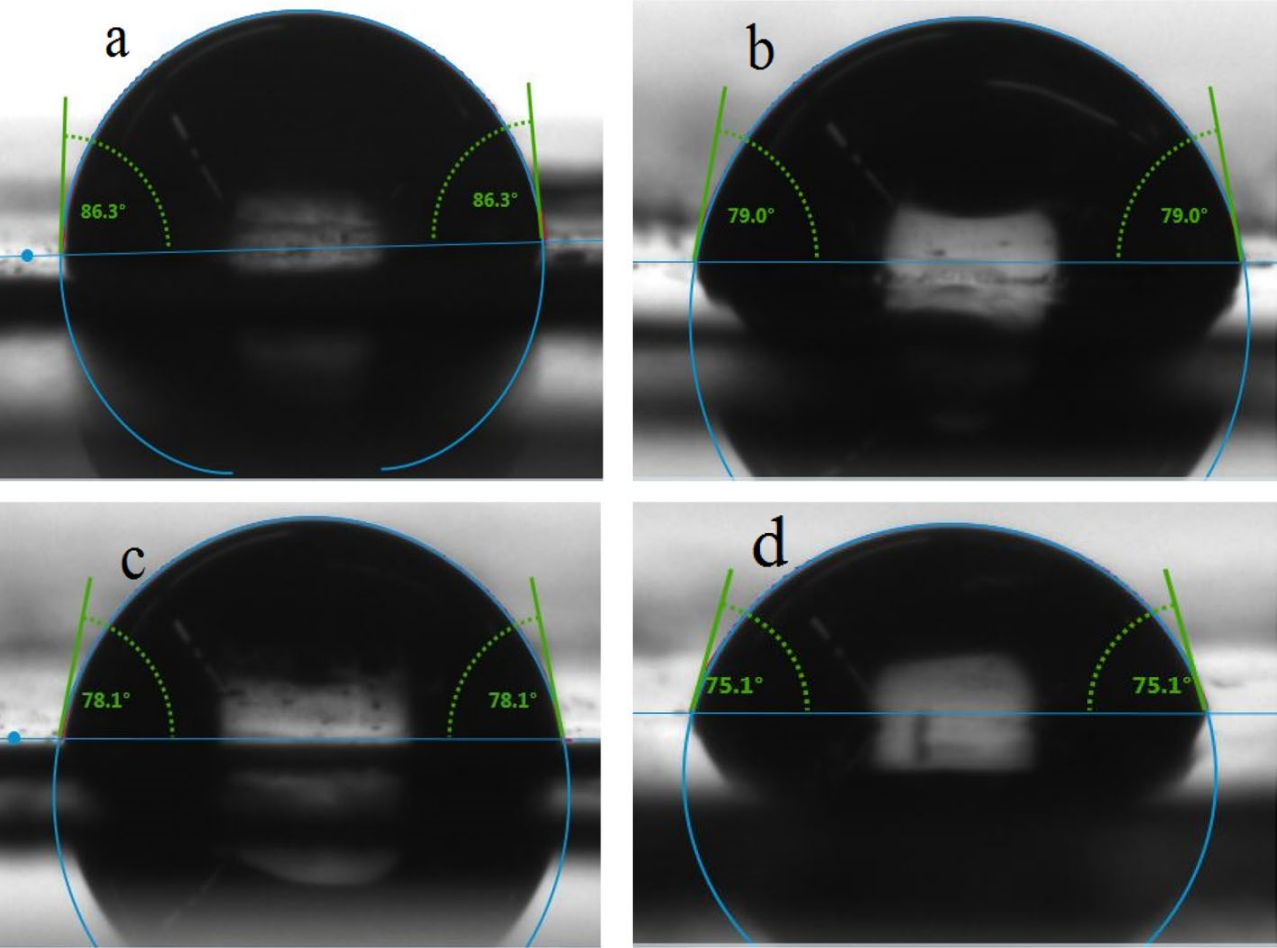
Contact angle of the fabricated PS/pTAP composite membranes, **(a)** PS/pTAP 100/0, **(b)** PS/pTAP 60/40, **(c)** PS/pTAP 50/50, **(d)** PS/pTAP 40/60.

The contact angles of the fabricated membranes were measured using the sessile drop method. The hydrophilicity of the PS/pTAP membrane changed after the addition of pTAP. With respect to the amount of pTAP added, the water contact angle observation showed a decreasing trend. The modified membrane with the addition of pTAP had a low water contact angle, with a contact angle of 75.1° for the 60% pTAP membrane, suggesting superior hydrophilicity. Figure 5 shows that the contact angles of PS/pTAP membranes decreased, suggesting that the addition of pTAP polymer greatly improved the AEMs hydrophilicity.

### Alkaline stability

The acceleration alkaline stability test was conducted by immersing the membrane in 1 M NaOH at ambient conditions for 300 h, and after that the property of ionic conductivity was measured and we observed that there is no much appreciable change in the ionic conductivity values. The values of ionic conductivity decreased by less than 2%, i.e. from 1.818 mS/cm to 1.787 mS/cm, and the same trend was also observed with the weight loss measurements which was resulting in less than 2% loss. Hence, these membranes possessed good alkaline stability and proved to be a viable candidate for working in any alkaline based EES applications.

### Permeability studies of fabricated composite membranes

The key advantage of AEMs is that they prevent active species cross-mixing by blocking the vanadium cation in RFBs through the Donnan exclusion effect, allowing for the use of more concentrated liquid fuels and thus improving overall system performance. In standard RFB systems, the permeability of vanadium ions plays a direct role in columbic efficiency and self-discharge rate.

**Figure 6:**
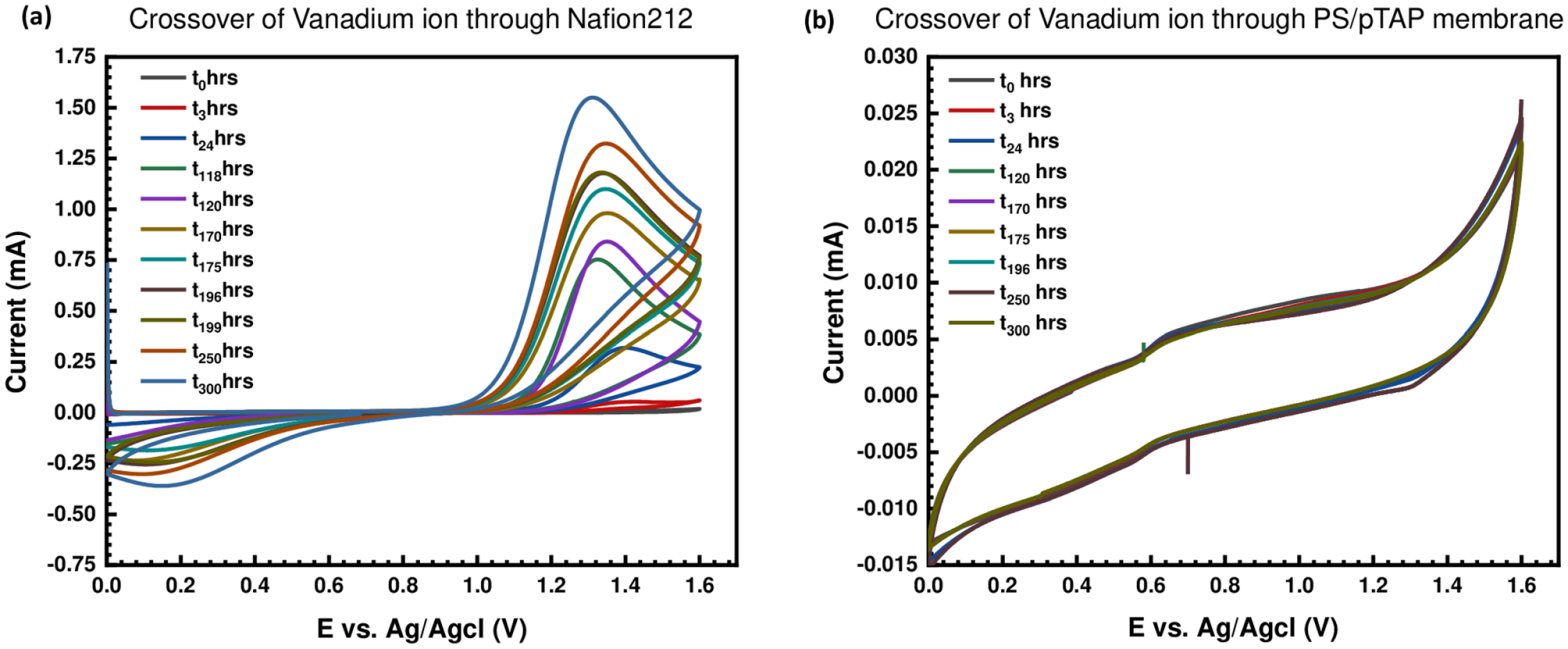
**(a)** Cyclic voltammogram for the crossover of vanadium through Nafion-212, and **(b)** PS/pTAP 40/60% composite membrane (right).

**Figure 7:**
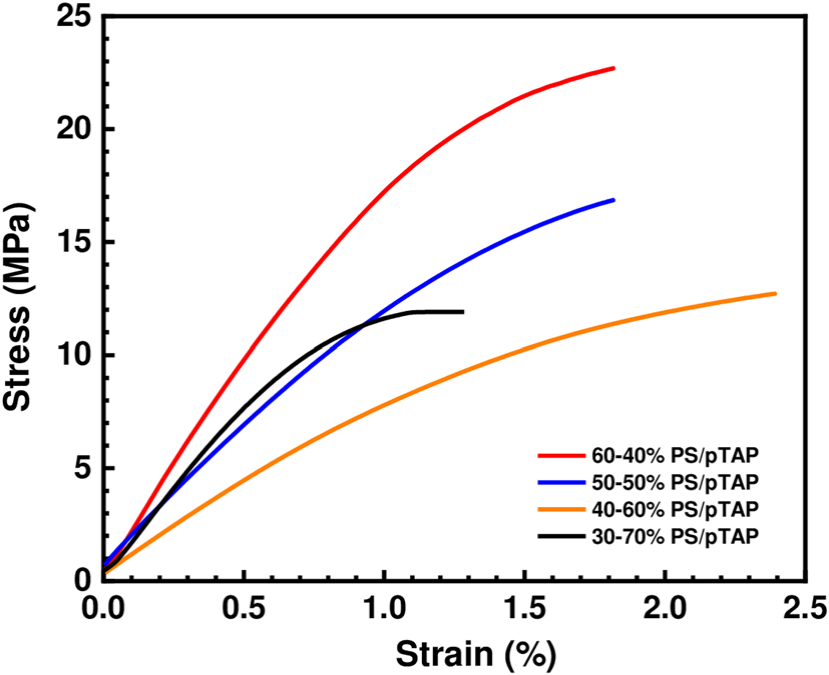
Mechanical stability of PS/pTAP composite blend membranes.

The vanadium permeability of the fabricated PS/pTAP 40%/60% membrane and Nafion-212 was investigated in this study using a two compartment cell to evaluate the vanadium ion crossover. Since the thickness of the membranes has a strong impact on the vanadium ion permeability measure, we used a membrane with a thickness equal to Nafion-212 for comparison. Because of the low swelling behavior and repulsion between the vanadium cation and the fixed cationic functional group in the AEM, as defined by the Donnan exclusion effect, the concentration of vanadium through the PS/pTAP membrane was almost negligible compared to that through the Nafion membrane (1.256 mmol/h). Due to the extremely low water absorption activity, the low IEC value, and the low Donnan effect, no active material permeation was observed at the diffusion cell with the PS/pTAP ionomer membrane within 300 h (Fig. 6). The vanadium ion crossover at different time intervals through Nafion-212 and the fabricated pTAP composite blend membrane is shown in Fig. 7.

### Mechanical strength

Although having a large number of functional groups on the polymer repeating unit can lead to improved efficiency, it can also lead to a loss of mechanical strength due to increased water absorption and micro voids between the polymer chains. To function optimally, a membrane must have both high mechanical stability and hydroxide conductivity. Tensile test results (see Fig. 7) showed that the tensile strength of the PS/pTAP membranes was significantly improved compared to that of the pure Nafion membrane. The commercial Nafion 212 membrane exhibited a tensile strength value of 9.0 MPa^35^, which is 3.7 times lower than for the best composite membrane fabricated in our work. The ultimate tensile strength of the fabricated composite membranes are found to be in the range of 11.8 to 22.5 MPa which is comparable with that of the already reported AEMs^36^. The PS/pTAP (60%/40%) showed the tensile strength and elongation-at-break of 22.5 MPa and 1.8%, while the PS/pTAP (40%/60%) showed that of 12.5 Pa and 2.5%, respectively. This showed that pTAP addition could improve the elongation-at-break and decrease the tensile strength of the PA/pTAP membrane. With the increasing of pTAP content from 40 to 70 wt. %, the tensile strength of the PS/pTAP membrane decreased from 22.5 to 11.8 MPa. The elongation-at-break of the PS/pTAP membrane increased from 1.5 to 2.5% with the pTAP ionomer content increased from 40 to 60 wt. %. However, when the pTAP content exceeded 60 wt. %, the elongation-at-break as well as tensile strength values of the PS/pTAP membrane decreased with the pTAP concentration. The enhanced elongation-at-break and reduced tensile strength of the PS/pTAP membrane could be attributed to the appropriate amount of pTAP, which accelerated the rate of plasticization of the PS/pTAP membranes. However, the excess pTAP would have an adverse effect on the mechanical properties of PS/pTAP membrane. Therefore, the concentration of pTAP in the PS/pTAP membrane should not exceed 70 wt.%.

### Thermal stability

**Figure 8:**
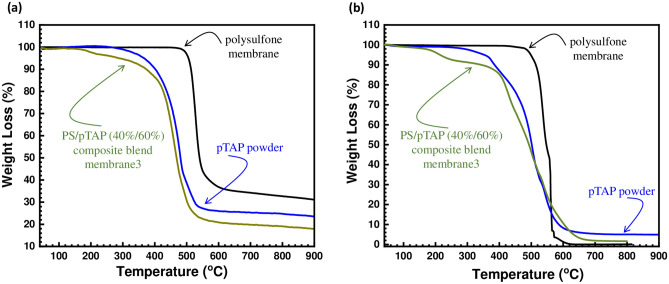
Thermal stability of polymeric based on polysulfone membrane; pTAP powder; and PS/pTAP (40%/60%) composite blend membrane in **(a)** in inert atmosphere and **(b)** oxygen atmosphere.

The fabricated composite blend membrane was tested by thermogravimetric analysis under argon as well as oxygen atmospheres to evaluate its thermal stability (Fig. 8). Polysulfone membrane was stable up to 400 °C in argon as well as oxygen atmosphere. The synthesized PS/pTAP composite membrane was stable up to 360 °C in nitrogen atmosphere without any major weight loss and 4–5 wt. % loss is observed in oxygen atmosphere after 200 °C which arises due to the elimination of solvent and water molecules. The major weight loss observed above 360 °C and 400 °C is due to the main chain degradation of the polymers. The thermogram showed that there is no significant weight loss observed for the –OH converted PS/pTAP composite membrane up to 200 °C and the 3–4 wt.% loss observed might be due to the elimination of water and solvent molecules. However, after 200 °C the weight loss corresponds to the degradation of phosphonium groups attached to the aryl chain. From the thermogram, it is inferred that the polysulfone is highly thermally stable up to 500 °C, compared to pTAP polymer which is stable up to 350 °C. Hence, when the pTAP polymer is added to the polysulfone to form composite blend membrane, its thermal stablility was found to be less than that of the pristine polymers.

It is expected that the weight loss observed above 350 °C results from decomposition of the polymer backbone. In general conclusion, we can ensure that our polymer in form of blend with polysulfone or alone can be stable at temperature below 200 °C in both argon and oxygen atmosphere. This is very promising data as the intended applications of such membrane in electrochemical energy devices at temperatures ranged from 50 to 150 °C.

### Morphology and surface topography analysis

**Figure 9:**
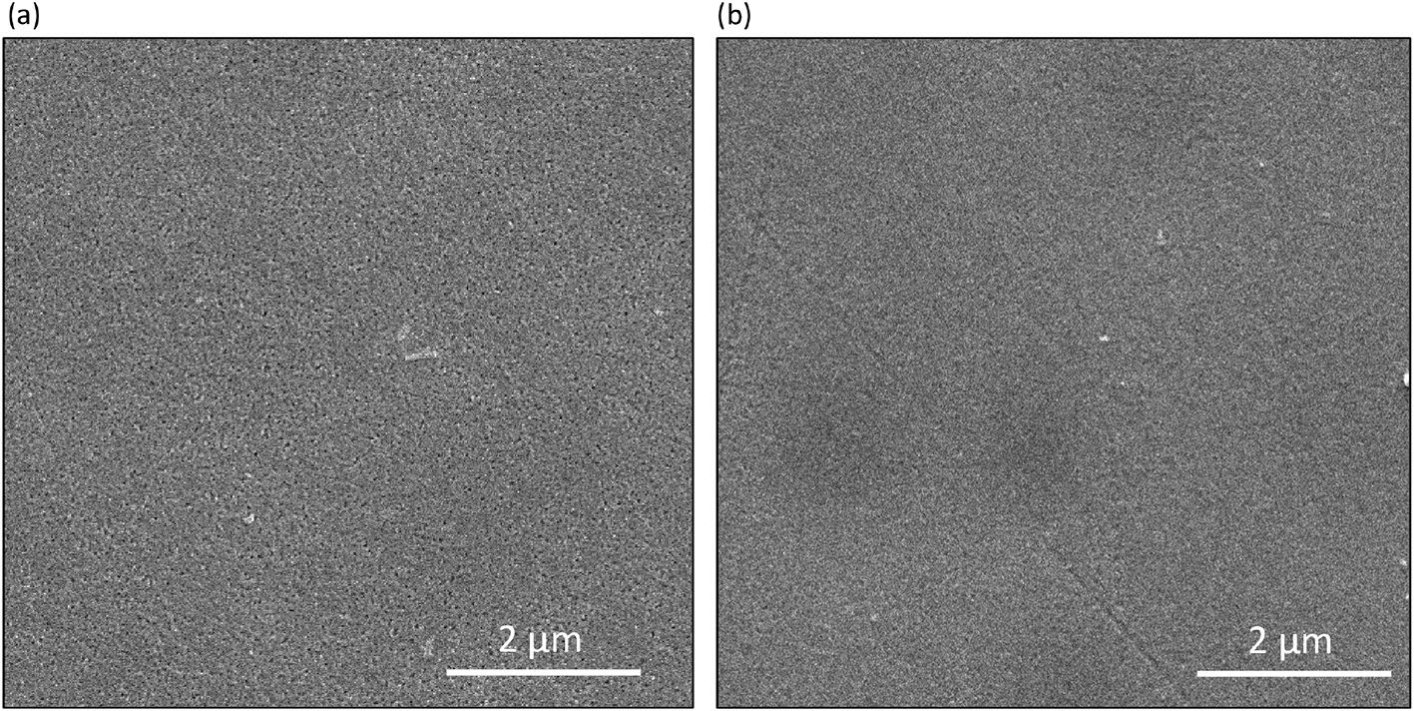
SEM images for **(a)** PS/pTAP (50%/50%) composite membrane, and **(b)** PS/pTAP (40%/60%) composite membrane.

**Figure 10:**
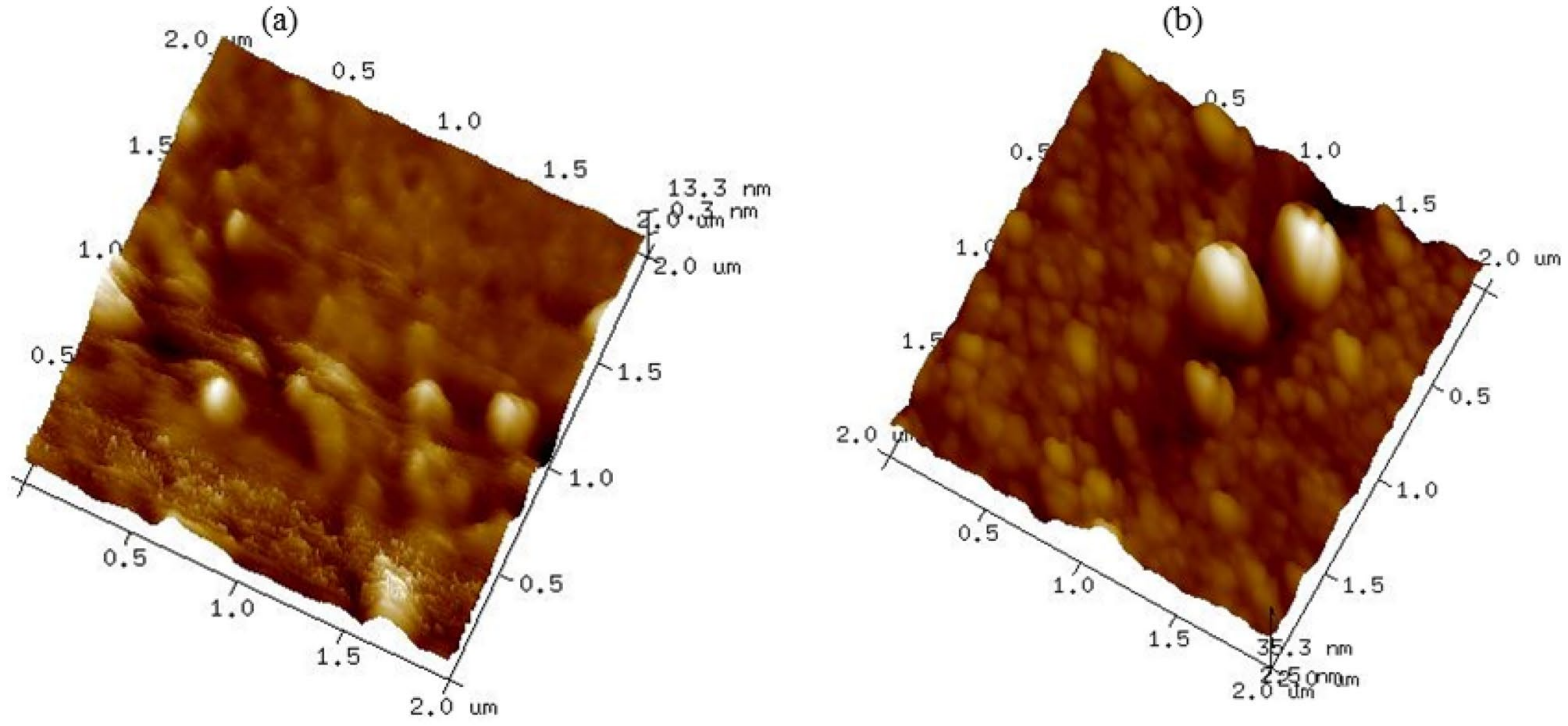
AFM images for **(a)** PS/pTAP (50%/50%) composite blend membrane, and **(b)** PS/pTAP (40%/60%) composite blend membrane.

The morphology of the prepared composite membranes PS/pTAP was investigated by scanning electron microscopy and it is shown in Fig. 9. The dispersion of pTAP polymer in the polysulfone matrix plays a significant role in influencing the physical properties of the membrane. The surface view of the composite blend membranes shows a homogenous and closed-packed structure in the morphology without any obvious phase separation or surface defects indicating the excellent phase compatibility of the pTAP polymer with the polysulfone matrix. The polymer–polymer distribution and polymer–polymer matrix reinforcement play vital roles for both compatibility and mechanical properties of the composite blend membranes. SEM analysis confirmed that the composite blend membranes up to 60 wt.% of pTAP ionomers are suitable for AEM based systems. Figure 10 indicates AFM 3D images of the PS/pTAP (50%/50%) and PS/pTAP (40%/60%) composite blend membranes. The thickness of the blend membranes was found to be uniform, with no noticeable change, resulting in improved adhesion properties during membrane and electrode assembly fabrication for electrochemical energy conversion/storage systems. The variations in domain hardness are shown by the bright and dark regions in the images. The hydrophobic polymer backbone was assigned to the bright regions, while the dark regions were assigned to a soft structure, which represented the hydrophilic ionomer groups^37^. As the weight percentage of ionomer in the composite blend was increased, the mean roughness of the membranes has increased by ~ 1300%, i.e. 0.096 nm to 1.23 nm, indicating the development of a homogenous blend associated with an improvement in the water uptake capacity of blend membranes.

### XRD of fabricated composite membranes

**Figure 11:**
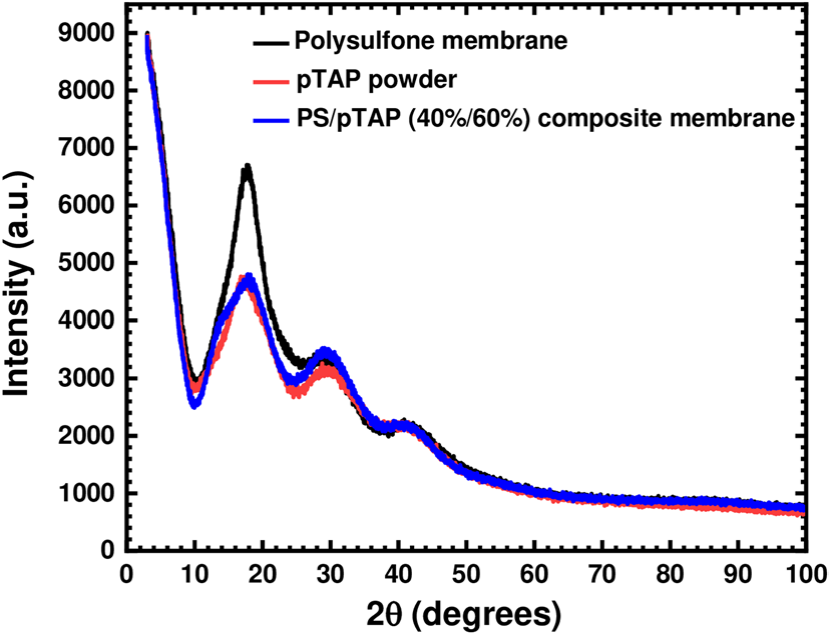
XRD spectra for polysulfone, pTAP polymer and the PS/pTAP composite membrane.

Figure 11 shows the XRD spectrum of polysulfone (PSF), which shows a wide, weak diffraction peak located at a 2ϴ value of approximately 17°, suggesting an almost amorphous structure for PSF. The XRD pattern of the composite membrane material with 60% pTAP had similar characteristic peaks as pure PSF, but there was a slight broadening of the peak, indicating that the incorporation of pTAP polymer within the polysulfone matrix causes a slight increase in polymer chain disorder. As a result, the composite membrane materials have a more amorphous form, which provides a pathway for ionic conduction in the composite membrane.

In summary, the composite membranes based on the pTAP polymer and PS were prepared by solution casting method. Due to the loss in mechanical properties the threshold weight percentage of pTAP content in PS matrix was limited to 60 percent. The thermal and mechanical analysis of the composite membrane revealed good thermal stability up to 200 °C and adequate mechanical features. From the XRD pattern, it has been observed that the crystallinity of the PS membrane decreased with the addition of pTAP polymer and makes the composite amorphous in nature. Our investigation shows that the composite membranes possess good ionic conductivity and water absorption properties, which make them suitable to work in alkaline environment. The PS/pTAP 40/60% membrane resulted in a maximum ionic conductivity of 4.2 mS/cm at 40 °C and negligible vanadium permeability over 300 h. Altogether, the preliminary analysis here reported on these PS/pTAP composite membranes have demonstrated that they could be potential candidates as polymeric electrolyte membranes in EES applications operating under standard alkaline conditions.

## Methods

All the reagents and solvents were purchased from Sigma-Aldrich, with Analytical grade, and used as received. The tetraarylphosphonium polymer used in our study has been prepared accordingly with literature procedure^19^. NMR spectra were recorded on a Bruker AVANCE III HD 300 MHz NMR spectrometer. NMR data were reported by using Aspin—NMR Data Reporting Tool^38^. The homogenous polymeric solution was cast on a glass tray using a doctor blade, and the thickness of the cast film was adjusted to 60 µm. The membranes were fabricated by the solvent evaporation technique. After casting, the film was dried in a vacuum oven at 90 °C for 24 h. After cooling at room temperature, the membrane was peeled off from the glass plate by immersing it in cold DI water. After then it was converted to –OH form by immersing them into 1 M KOH solution for 24 h at ambient conditions. Immersion in KOH is expected to lead to a hydroxide ion exchange membrane [pTAP^+^][OH^−^]. This step has been repeated three times to ensure a complete anion exchange. Fourier-transform infrared (FTIR) spectroscopy was performed to confirm the presence of functional groups in the synthesized pTAP polymer and as well as its possible interactions with the polysulfone matrix in the composite blend membranes. The FTIR spectrometer (Alpha Bruker spectrometer) was used for analysis and the spectra were measured over a wave range of 4000–500 cm^−1^ in absorbance mode by placing the membranes in potassium bromide window. The ion exchange capacity (IEC) was assessed by the titration method. The dried membrane in the hydroxyl form was immersed in 3 M sodium chloride solution for 48 h at room temperature. The –OH^-^ ions, substituted by Cl^-^ ions, were released into the solution. The solution was then titrated by a 0.01 M HCl solution using phenolphthalein as an indicator. The IEC of the membranes were calculated using the following equation^39^:

1 $${\text{IEC = C}}_{{{\text{HCl}}}} \times {\text{ V}}_{{{\text{HCl}}}} /{\text{ W}}_{{{\text{Dry}}}}$$

where C _HCl_ (mol/l) and V _HCl_ (ml) are the concentration and volume of HCl solution required for the neutralization of the residual solution, and W_Dry_ is the weight of the dry sulfonated membrane.

The conductivity of the membranes in –OH form was measured by AC impedance technique using a Biologic Electrochemical Analyzer. Membrane samples were equilibrated in 1 M KOH for 48 h at room temperature before testing. Then the membranes were blot dried with tissue paper and sandwiched between two graphite electrodes of the conductivity cell. The impedance spectra were recorded over the frequency range of 7 MHz with 10 mV oscillating voltage. The membrane conductivity was calculated from the impedance data, using the relation^40^:

2 $$\sigma {\text{ }} = {\text{ L}}/{\text{RS}}$$

where L and S are the thickness and area of the membrane and R can be derived from the low intersect of the high frequency semicircle on a complex impedance plane with the Re (Z) axis.

Water uptake measurements were carried out by measuring the change in weight of the membrane before and after hydration. The − OH form of the membrane was immersed in deionized water at room temperature and equilibrated for about 24 h. The wet weight of the membrane was determined after removing the excess surface water. The percentage of water content was calculated using the following relation^41^:

3 $${\text{Water uptake }}\left( \% \right) = {\text{ 1}}00{\text{ }} \times {\text{ }}\left[ {{\text{Weight of the wet polymer }} - {\text{ Weight of the dry polymer}}} \right]/{\text{ Weight of the dry polymer}}$$

Water contact angle measurement was measured by sessile drop method, with 250-F1 goniometer (Rame-hart Instrument Co, USA) and used to examine the change in hydrophilicity of the fabricated composite blend membranes^42^. The contact angle measurement was then performed as follows. A dangling droplet of 6 μL of DI water at the end of ‘I’-shaped needle was carefully deposited to membrane surface to avoid the effect of falling force by gravity. Three measurements were taken for each membrane and the average contact angle has been reported. The alkaline stability of the membranes was measured by immersing the sample in 1 M sodium hydroxide solution for 300 h^43^. The alkaline stability test (or durability test) was performed to analyze the durability of the membrane in the real cell condition. Cyclic voltammetry was used for measuring the vanadium permeability of the membrane. Experiments were carried out at ambient temperature in a two compartment glass cell (with a capacity of 100 ml) with membrane placed in between, to separate the two half cells. A glassy carbon electrode and smooth platinum electrodes were used as working and counter electrodes, whereas Ag/AgCl was used as a reference electrode. The electrolyte used was 2 M vanadium sulfate in 2 M sulfuric acid. The vanadium solution was introduced into the left side of the two compartment cell, and on the right side 2 M sulfuric acid solution was placed. The vanadium permeation from side1 to side 2 through the membrane was observed from the vanadium oxidation currents measured through cyclic voltammetry at various time intervals. The vanadium concentration and diffusion coefficient were determined from the measured transient limiting current density following a potential step. The vanadium flux was established across the membrane due to the concentration difference between the compartments. The vanadium flux was found to be dependent on the state of hydration and on the thermal history of the membrane. The concentration of vanadium in the receiving compartment as a function of time has been calculated as previously reported in the literature^44,45^. The mechanical tensile tests were performed according to the standard method using universal testing machine (Hounsfield Universal Testing Machine). The samples were cut into a size of 5 mm × 50 mm wide strips and the thickness of each strip was measured with a digital micrometer. The tensile strength of the prepared ion exchange membrane was directly obtained from the tensile tests^45–48^. The thermal stability of the composite blend membrane was examined by the thermogravimetric analyzer (Netzsch Thermal Analyzer). About 10 mg of the sample was used. The experiment was scanned over temperatures between 300 °C and 800 °C under nitrogen as well as in the oxygen atmosphere, at a heating rate of 10 °C/min. The SEM micrographs of the PS/pTAP composite membranes were obtained with the help of Bruker D8 Advance X-ray diffractometer. The AFM 3D topography images of the PS/pTAP composite blend membranes were obtained with Bruker Dimension Icon AFM.

## Supplementary Information

Supplementary Information.
