# Supplementary material for: Investigating the suitability of poly tetraarylphosphonium based anion exchange membranes for electrochemical applications
Source: Sci Rep. 2021 Jul 5;11:13841. doi: 10.1038/s41598-021-93273-x (PMC8257644; doi:10.1038/s41598-021-93273-x)
Supplement: Supplementary file 1 — Supplementary Information. [file 41598_2021_93273_MOESM1_ESM.docx]

**Supplementary Material**

**Investigating the suitability of poly tetraarylphosphonium based anion exchange membranes for electrochemical applications**

Muthumeenal Arunachalam^1^, Alessandro Sinopoli^1^_,_ Farida Aidoudi^1^, Stephen E. Creager^2^, Rhett Smith^2^, Belabbes Merzougui^1^ & Brahim Aïssa^1*^

*^1^Qatar Environment & Energy Research Institute, P.O. Box: 34110, Doha, Qatar*

*^2^* *Department of Chemistry and Center for Optical Materials Science and Engineering Technology, Clemson University, Clemson, South Carolina, USA*

**Corresponding authorS*ax: +974 4454 41528, Phone: +[974 4454 7475](tell:+97444547475)

*Synthesis and NMR characterisation of pTAP polymer.*

The requisite 4,4'-(ethane-1,2-diylbis(oxy))diphenyl bistriflate (1.372 g, 2.69 mmol), diphenylphosphine (0.525 g, 2.82 mmol), diisopropylamine (0.274 g, 2.69 mmol), Pd_2_(dba)_3_ (0.0270 mmol, 0.0280 g) and 6 mL anhydrous ethylene glycol were mixed together in a 15 mL heavy-wall pressure tube sealed with a Teflon stopper and Viton O-ring under a nitrogen atmosphere. The sealed vessel was heated at 145 °C with stirring for 24 h. After cooling down to room temperature, the reaction vessel was opened to air and 50 mL DCM was added. The mixture was then washed with 0.5 M LiOTf(aq) (2×50 mL). The addition of triflate anions in the aqueous phase was undertaken as a precaution against any adventitious anion exchange. The organic layer was dried over anhydrous MgSO_4_ and concentrated to 10 mL under reduced pressure by rotary evaporation. The crude solution was slowly poured into 200 mL diethyl ether with stirring to yield a cloudy suspension. The solid was separated by vacuum filtration and dried in a vacuum oven overnight at 60 °C to give the desired product. Yield: 72.5%.

^1^H NMR (300 MHz, (CD_3_)_2_CO) δ: 8.10–7.52 (br m, 18H), 4.44 (s, 4H); ^31^P NMR (121MHz, (CD_3_) _2_CO) δ: 25.3-25.7 (phosphine oxide end group), 23.99 (main phosphonium peaks); ^19^F NMR (282 MHz, (CD_3_) _2_CO) δ: -78.72 (main backbone), -74.15 (end group-associated triflate).

Figure S1: Proton NMR spectrum of pTAP polymer ((CD_3_)_2_CO, 300 MHz). The spectrum shows signals corresponding to water and hexane solvent impurities.

Figure S2. Phosphorus NMR spectrum of pTAP polymer ((CD_3_)_2_CO, 121 MHz).

Figure S3. Fluorine NMR spectrum of pTAP polymer ((CD_3_)_2_CO, 282 MHz).
